# “High‐risk” tumors of the lip treated with external beam radiotherapy and high‐dose‐rate brachytherapy: Long‐term outcome

**DOI:** 10.1002/hed.27936

**Published:** 2024-09-26

**Authors:** Claes Mercke, Signe Friesland, Anders Berglund, Gun Wickart Johansson, Gregori Margolin, Michael Gubanski, Einar Björgvinsson, Josef Nilsson

**Affiliations:** ^1^ Department of Head, Neck, Lung and Skin Cancer, Theme Cancer Karolinska University Hospital Stockholm Sweden; ^2^ Department of Oncology‐Pathology Karolinska Institutet Stockholm Sweden; ^3^ Epistat Epidemiology and Statistics Consulting Uppsala Sweden; ^4^ Division of ENT Diseases, Department of Clinical Sciences, Intervention and Technology Karolinska Institutet Stockholm Sweden; ^5^ Department of Radiation Oncology Karolinska University Hospital Stockholm Sweden; ^6^ Department of Medical Radiation Physics and Nuclear Medicine Karolinska University Hospital Stockholm Sweden

**Keywords:** brachytherapy, head and neck surgery, lip cancer, oncologic outcomes, radiotherapy

## Abstract

**Background:**

Radiotherapy is a well‐established treatment for lip cancer, with external radiotherapy (EBRT) or brachytherapy (BT).

**Methods:**

This study evaluated outcome, tumor control, and aesthetics, for 101 patients with carcinoma of the lip, not suitable for surgery, treated with combined EBRT and BT.

**Results:**

Squamous cell carcinoma was seen in 78 patients, basal cell carcinoma in 15, and other histologies in 8 patients. Tumors were advanced: 73% in category T2‐T4. Local control at 3 and 5 years was 89%. Local failure appeared in 4/56 patients (7%) with primary RT compared to 7/45 (16%) in those with prior surgery, regional recurrence in 5 patients. Toxicity was mild. Cosmetic outcome, 87 patients evaluated, was bad for 9/40 patients with upfront surgery compared to 1/47 for primary RT patients (*p* = 0.003). Seven patients died from lip cancer (7%), three with originally N+ disease (43%).

**Conclusions:**

Combined EBRT and BT could be considered for lip tumors not candidates for surgery.

## 1 INTRODUCTION

Cancer of the lip belongs to those tumors of the face which are most challenging to treat when it comes to balance the control of disease with a satisfactory functional and cosmetic outcome. Focus on treatment outcome is on tumor eradication but the preservation of function and cosmetics is essential to guarantee the patient adequate nutrition and communication with people. The most common histological tumor type is squamous cell carcinoma (SCC) followed by basal cell cancer, but also carcinomas of other histology can arise in the lips.

Different therapeutic options are available for patients with lip carcinomas, surgery, external beam radiotherapy (EBRT), and brachytherapy (BT). The majority of lesions not exceeding 2.0 cm, that is, T1 tumors, are excised surgically with primary closure as an outpatient procedure and is chosen by the surgeons. The results of such an intervention are often considered satisfactory as long as the oral commissure is not resected and if the resulting aperture of the oral cavity permits a proper insertion of dentures.^1^ The procedure is also rapid with a short postoperative healing time. However, long‐term cure rate for these smaller tumors is similar for surgery and EBRT.^2^

For moderately advanced lesions, T2 tumors of 2–4 cm, removal of greater than half of the lip with simple closure can produce a poor cosmetic and functional result so that a reconstructive procedure is considered necessary. Therefore, primary treatment for such tumors has in several institutions relied on radiotherapy (RT) which is also common for T3 and T4 tumors.

Considerable experience has been collected with low‐dose‐rate brachytherapy (LDR‐BT), performed with Radium or Cesium needles as well as with Iridium wires in the treatment of lip cancer during the last century. Such treatment has yielded excellent local control rates and aesthetics and has been executed without major toxicity.^3^, ^4^ LDR‐BT is no longer available but has been an established treatment for lip cancer with 5‐year local control rates of 90%–95%.^5^ When Iridium wires were no longer available on the market, high‐dose‐rate (HDR‐BT) and pulsed‐dose‐rate brachytherapy (PDR‐BT), were introduced with the possibility to improve patient comfort, optimize implant homogeneity and decrease radiation exposure to the staff. Most suggest results comparable with LDR‐BT.^1^, ^6^, ^7^

The choice of therapeutic regimen for patients with newly diagnosed lip carcinomas in our institution is taken at multidisciplinary conferences with specialists in head and neck surgery, plastic surgery, diagnostic radiology, pathology, and oncology. To optimize treatment methods, HDR‐BT was introduced for patients with lip cancer in 2002 in our center, meant to be a main component of a standard program for the sterilization of the local disease in the lip.

The aim of the present analysis is to explore long‐term results of a single‐institutions' experience with a proposed standard treatment program for patients with malignant tumors of the lip vermillion, irrespective of histological subtype, focusing on local control of the lesion in the lip, function, and cosmetic outcome. The aim of the study is also to define what patients with lip cancer for whom the proposed schedule was changed in order to adequately treat the individual patient's disease. Based on the findings of the present analysis the potential role of interstitial HDR‐BT as an option for primary treatment of all carcinomas of the lip will be discussed. The present study was approved by the Swedish Ethical Review Authority.

## 2 METHODS

### 2.1 Patients

We examined retrospectively the clinical records of all consecutive patients treated by BT from January 2002 to December 2022 at our institution (Department of Oncology, Karolinska University Hospital, Stockholm, Sweden) for a histologically confirmed invasive carcinoma of the lip. Patients were staged according to the TNM system (TNM 7), underwent mandatory blood tests, had CT and/or MRI examination of the oral cavity and neck for assessment of regional lymph nodes, a lesion biopsy for tumor pathology and if indicated a fine needle aspiration of neck nodes. Performance status (PS) according to the ECOG scale was used to quantify the functional status of the patient. Indications for treatment according to the standard program were as follows: (1) as primary treatment for patients for whom surgery could be mutilating; (2) for patients with positive resection margins after surgical excision or with local recurrence soon after primary surgery.

### 2.2 The RT regimen/the standard program‐general considerations

Many lip carcinomas are exophytic, protruding from the lip surface. To cover this tissue with BT, needles are sometimes added with a template outside the lip.^8^, ^9^ The rationale to start with EBRT in this regimen was twofold: (1) To shrink the exophytic component to secure adequate coverage of the tumor with BT; (2) to diminish the potential negative impact of the high‐dose 150% and 200% volumes of BT when the intended curative RT dose was reached. Therefore, the standard program stipulated that treatment started with EBRT, delivered with 3.0 Gy per day in 10 fractions, followed by HDR‐BT in 7–8 fractions of 3.0 Gy twice a day, at least 6 h apart. This combined RT dose corresponded to a biologically effective dose BED_10_ of 66–70 Gy. Adjuvant RT to the N0 neck was not stipulated. This standard program was meant to be considered for all patients. It was anticipated that modifications would be necessary due to individual tumor and patient characteristics.

### 2.3 External beam radiotherapy

The gross tumor volume, GTV was contoured based on physical examination of the lip and neck and on radiological findings with CT or MR. CTV was defined as GTV with a suitable margin and with an additional margin of approximately 5 mm to PTV. Bones, including the mandible, the spinal cord and the oral cavity were contoured. Identical target definitions were used for tumors remaining after a surgical procedure albeit sometimes for smaller volumes.

Neck nodes in level I could be included in the target for bulky T3/T4 tumors as well as level II for tumors approaching the buccal mucosa. The most frequent radiation technique was with two opposed radiation fields.

When a patient had neck node metastases, the treatment program was adjusted accordingly and combined with lymph node dissection and/or external beam radiotherapy (EBRT) treating the neck. EBRT given postoperatively or as radical therapy was taken to 68 Gy with nodes in level I‐IV in the target for N1‐N3 disease.

### 2.4 Brachytherapy

Brachytherapy (BT) started 7 days after EBRT. The implantation procedure was executed in an operating theater with the patient under general anesthesia. Hollow rigid trochars were inserted by the radiation oncologist across the length of the tumor (the GTV), aimed at a distance between the needles of 8–12 mm, placed horizontally in a parallel fashion in order to cover the macroscopic tumor plus 2–5 mm, constituting the CTV. For most tumors parallel needles were placed in an equilateral triangular arrangement and for larger lesions, additional needles could be added. In not well demarcated tumors, the safety margins around GTV could be even more than 5 mm and sometimes the whole lip could be included. The rigid steel needles were replaced by plastic tubes, secured in position using plastic buttons. After the insertion of BT catheters, a dose planning CT scan with a slice thickness of 2.0–2.5 mm was acquired to define the CTV. An individual three‐dimensional dose plan was generated (Plato BPS, Nucletron, The Netherlands, or Oncentra Brachy, Elekta, Sweden) and exported to the afterloader (HDR or PDR microSelectron, Flexitron, Elekta, Sweden). A physical dose of 24 Gy was prescribed but could be 21 Gy for small clinical target volumes. The implantation of catheters was performed on Monday, BT pulses delivered daily from Tuesday during the rest of the week and catheters extracted on Friday. So, BT could be completed in less than 1 week. Most patients had their BT delivered through OncoSmart catheter system, Elekta, Sweden. After completed treatment, plastic catheters were removed under mild sedation.

### 2.5 Dosimetric brachytherapy parameters

Dwell times were optimized with the intention to cover the CTV while V150%/V200% should approach 40/20. Dosimetric parameters, including V150% and V200% of the prescription dose, D90%, DNR, and COIN index, were recorded later. No dose constraints were used for organs at risk. However, by inserting sterile gauze dressings between the target volume and the mandible, this part of the oral cavity could be separated from the catheters, decreasing the dose to this tissue. The gauze dressing was changed before each fraction which made it possible to adapt its thickness related to the current shrinkage or swelling of the tumor.

### 2.6 Follow‐up, oncologic, and toxicity outcomes

Patients were first seen about 6 weeks after treatment. Patients had then follow‐up visits with clinical examination every 3 months for the first 2 years and every 6 months for another 3 years. Data of patient outcome, tumor control, and toxicity were collected from a local quality registry with prospectively gathered data, and supplemented with a review of medical records, including detailed data of delivered RT. Adverse effects, functional and aesthetic, were available from the registries. If tumor recurrence was suspected, a tissue biopsy and relevant radiological imaging was performed to define the recurrence as local, regional, or distant. At the same visits toxicity was graded according to the RTOG/EORTC scoring system for side‐effects after RT.^10^ Toxicities presenting later than 90 days after last day of irradiation were registered as late toxicities. Registered side‐effects were mucositis, soft tissue necrosis, fibrosis, xerostomia, osteoradionecrosis, oral dysesthesia, and trismus. Aesthetic outcome was labeled as “excellent” if no scar was visible, if lip retraction, atrophy, or telangiectasia were absent, as “good” if the same signs were slight, “fair” when they were more significant, and “poor” when lip retraction was pronounced, and/or atrophy, telangiectasia, asymmetry, or dyschromia was severe.

### 2.7 Statistical analysis

The primary endpoint of the study was local tumor control in the lip. Secondary endpoints include regional control, local recurrence‐free survival (LFS), disease‐free survival (DFS), toxicity, and cosmetic outcome. LFS was defined as the time from the last day of irradiation to the time of local disease progression or death and DFS as the time from the last day of irradiation to the time of disease progression locally in the lip, regionally in lymph nodes, at distant sites or death. Statistical analysis included overall survival (OS) defined as the time between the last day of RT to the time of death or the last day of clinical follow‐up. Recurrence of tumor was defined as first site of registered recurrence, local, regional, or distant.

Statistical analyses were performed to explore the impact of T category, size of tumor (size within respective T), tumor pathology, tumor grade (squamous cell carcinoma), effect of upfront surgery versus primary RT, tumor growth in oral commissure, growth pattern, age, and sex. All time‐to‐event endpoints were calculated using the Kaplan–Meier method and differences were tested using the log‐rank test. Statistical significance was considered with a test result below 5%, and analyses were performed using R version 3.6.1.

## 3 RESULTS

### 3.1 Patient characteristics and delivered treatment

During the study period 104 patients were treated in our institution for a cancerous lesion of the vermillion lip with a combination of EBRT and BT. Three patients were lost to follow‐up, one patient because of early death soon after inclusion, and two patients because they had moved, leaving 101 patients for the intention to treat (ITT) analysis. A majority of patients, 91% had a performance status (PS) of 0–1.

The mean and median follow‐up times were 7.2 and 5.9 years, respectively (range, 0.2–20.7). The patient with the very short follow‐up died from heart disease without signs of tumor recurrence or complications from treatment. There were 45 women and 56 men and median age was 71 years (range, 9–95). Smoking history was available for 71 patients (71%) with the majority being current or ex‐smokers (62%). Seventy‐eight patients had SCC, 15 basal cell carcinomas; 8 patients had other carcinomas, 4 salivary gland carcinomas, 2 Merkel cell carcinoma, 1 alveolar rhabdomyosarcoma, and 1 malignant melanoma. Differentiation of SCC could be evaluated in 62 out of 78 patients: 25 had well differentiated, 23 moderately differentiated, and 14 poorly differentiated tumors. The tumor was located in the lower lip in 77 of all patients (76%), in the upper lip in 24 (24%). Seventy‐one patients with SCC (91%) had their tumor in the lower lip. Forty patients (40%) had tumor involving the oral commissure, with a margin of <5 mm. Thirty out of the 78 patients with squamous cell carcinoma were characterized by such extensive growth (38%). Most tumors were medium‐sized to large, 73% in T2‐T4 and 53% larger than 3 cm. There were 27 patients with T1 tumors (27%), 54 with T2 (53%), and 20 with T3/T4 tumors (20%). Six patients had nodal metastases on presentation, 5 had squamous cell histology and 1 Merkel cell carcinoma, 5 patients with N1 and 1 with N2 disease. Detailed patient and tumor characteristics including actual tumor size within each T category and histology are summarized in Table 1.

**TABLE 1:** Patient and tumor characteristics.

Characteristics	Initial RT, *n* = 56 (%)	RT after curative surgery, *n* = 45 (%)
Age (years)		
Median	72	71
Range	9–95	34–91
Sex		
Female	21 (38)	24 (53)
Male	35 (63)	21 (47)
WHO performance status		
0	39 (70)	31 (69)
1	14 (25)	8 (18)
≥2	3 (5)	3 (7)
N/A	0 (0)	3 (7)
Site		
Lower lip	48 (86)	28 (62)
Upper lip	9 (16)	17 (38)
Oral commissure	25 (45)	15 (33)
Histology		
Squamous cell carcinoma	49 (88)	29 (64)
Basal cell carcinoma	3 (5)	12 (27)
Other^a^	4 (7)	4 (9)
Tumor classification		
T1	7 (13)	20 (44)
T2	33 (59)	21 (47)
T3‐T4	16 (29)	4 (9)
Tumor diameter (cm)		
≤1.5	5 (9)	14 (31)
>1.5 and ≤2	2 (4)	6 (13)
>2 and <3	8 (14)	13 (29)
≥3 and ≤4	25 (45)	8 (18)
>4 and ≤4.5	3 (5)	1 (2)
>4.5	13 (23)	3 (7)
Nodal classification		
N0	52 (93)	43 (96)
N1	3 (5)	2 (4)
N2	1 (2)	0 (0)
Tumor grade		
Well	17 (30)	8 (18)
Moderate	16 (29)	7 (16)
Poor	5 (9)	9 (20)
N/A	18 (32)	21 (47)
Growth pattern		
Exophytic	37 (66)	18 (40)
Infiltrative	46 (82)	38 (84)

^a^ Four salivary gland carcinomas, two Merkel cell carcinoma, one malignant melanoma, and one alveolar rhabdomyosarcoma.

In 56 patients out of 101 (56%) RT was given as primary therapy and in 45 patients (45%) after prior curative surgery.

**FIGURE 1:**
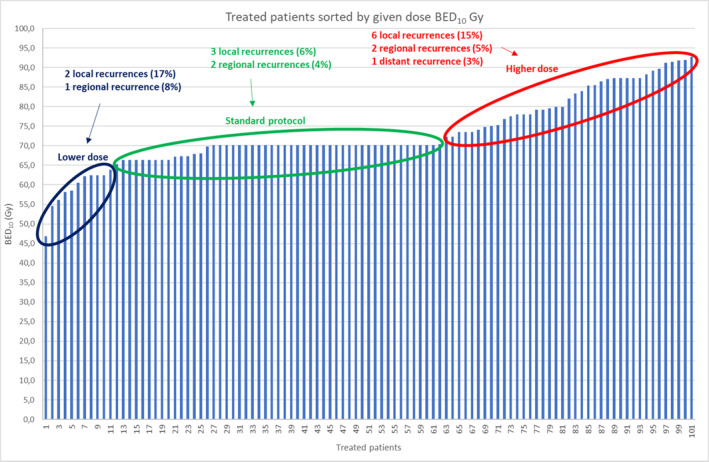
Given treatment and recurrences in relation to dose BED_10_ Gy. [Color figure can be viewed at wileyonlinelibrary.com]

**FIGURE 2:**
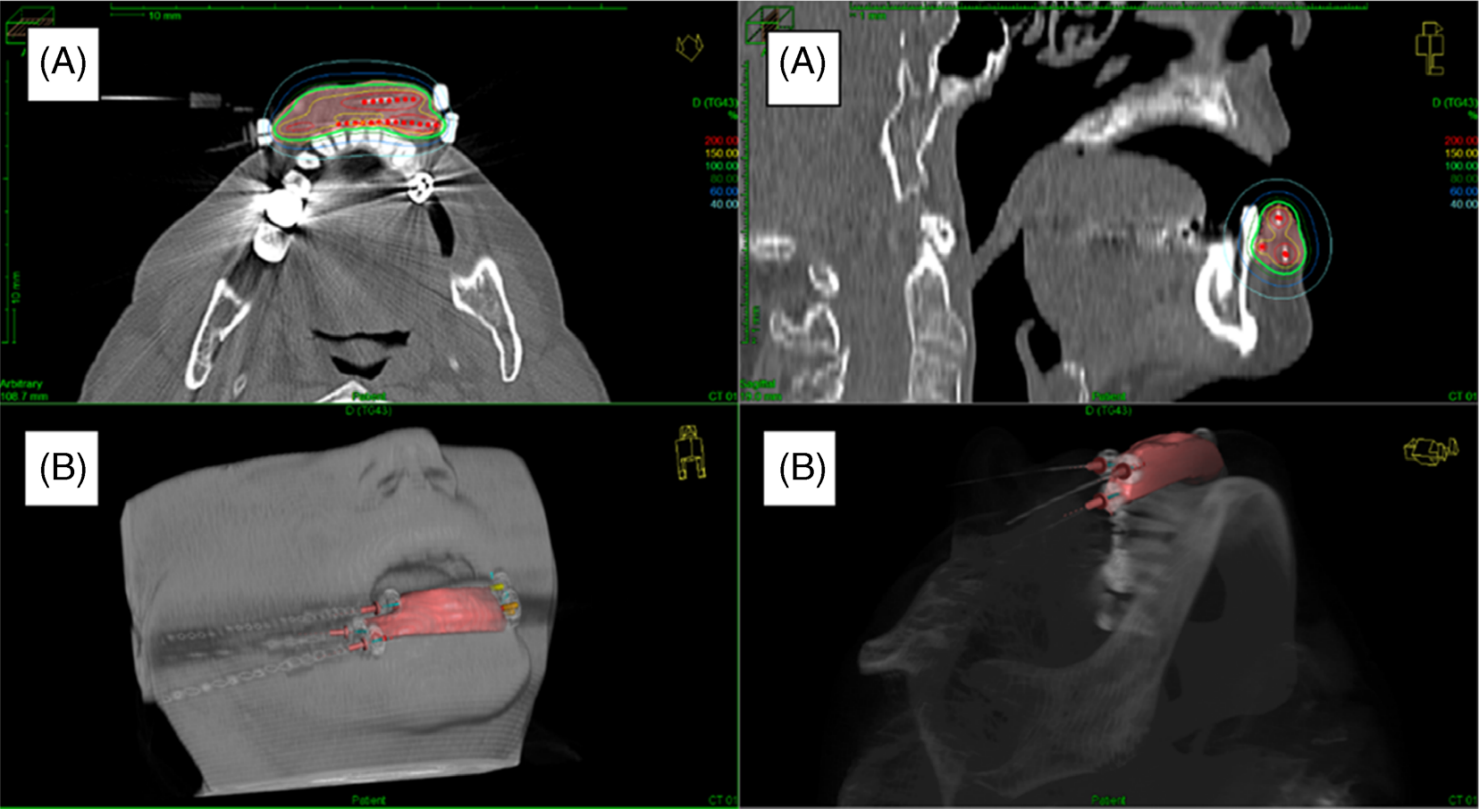
Example of brachytherapy treatment planning of the lower lip. (A) Dose distribution. (B) Implantation of three catheters inside clinical target volume (CTV) and sparing of the mandible. [Color figure can be viewed at wileyonlinelibrary.com]

Characteristics of delivered RT are shown in Table 2. Modifications of the standard protocol were undertaken with respect to BED, increased for large T2 tumors, T3 and T4 tumors, tumors of salivary gland histology and decreased for frail patients, and with respect to target volume for tumors growing close to the buccal mucosa, and for patients with positive neck nodes. The modifications of given doses BED_10_ Gy for all patients in relation to the standard program, lower (BED_10_ 46–65 Gy) or higher (BED_10_ 71–93 Gy), are displayed in Figure 1. A CT plan showing the coverage of tumor, the dose distribution, sparing of the mandible, and placement of the catheters can be seen in Figure 2.

**TABLE 2:** Characteristics of radiotherapy.

Characteristics	
Prescribed dose, median (range)	
EBRT (Gy)	30.0 (0–60.0)
BT (Gy)	24.0 (15–65.3)
Total prescribed dose (Gy)	54.0 (42.0–79.6)
Treatment time, median (range)	
EBRT (days)	14 (11–51)
BT (days)	4 (3–25)
Overall treatment time (days)	25 (8–119^a^)
CTV volume, median (range)	
EBRT (cm^3^)	20.2 (8.8–324.5)
BT (cm^3^)	9.3 (2.4–23.5)
Specific BT characteristics, median (range)	
Catheters (N)	4 (2–8)
V150 (cm^3^)	4.7 (1.4–15.1)
V200 (cm^3^)	2.1 (0.7–7.6)
D90%	92.6 (66.8–106.5)
DNR	0.49 (0.38–0.69)
COIN	0.66 (0.37–0.82)

^a^ Treatment interrupted and prolonged due to patient heart disease.

The standard protocol was given to 39 patients and to 34 patients with SCC, but additional 11 patients reached the same BED_10_ Gy. During the last 5 years from 2017 to 2022 more than 90% of patients selected for the regimen followed it in all respects.

### 3.2 Local tumor control

Eleven patients of all 101 patients (11%) experienced local recurrence as first site of relapse, 10 of the 78 patients with SCC (13%), 1 with a basal cell carcinoma and no patient with another histology. Local control at both 3 and 5 years was for all patients with T1 tumors 88%, for T2 tumors 91%, and for T3/T4 tumors 85% (*p* = 0.78). For patients with SCC respective figures were 89%, 87%, and 82% (*p* = 0.77). Six out of the 11 patients with a local recurrence, 5 with SCC, and 1 with basal cell cancer could be successfully salvaged, resulting in a final local control rate of 95% for all patients in the cohort and of 94% for patients with SCC. In the group of five patients whose disease could not be salvaged, two patients had a continuous propagation of their disease despite delivered therapy. Among the 11 patients with a local relapse, 4 were treated with EBRT and BT as their primary therapy, 1 patient with a T4 tumor, and 3 with large T2 tumors, whereof 2 N+. Seven patients had prior surgery before their RT, 2 with a T3, 2 with T2, and 3 with T1 tumors, whereof 1 was large. This means that local failure was twice as common in patients who had prior surgery, in 7/45 patients (15.6%) compared to those who started with RT where failure was seen in 4/56 (7.1%). For the homogenous group of patients with SCC, considered appropriate to follow the standard program precisely, there were 34 patients, 19 with the regimen as their primary treatment, and 15 patients treated after attempted curative surgery. There were no local failures (0/19 = 0%) in the former group and three failures (3/15 = 20%) in the latter. One patient presented a new second primary on the other side of the lip 4.2 years after treatment with RT. Median time to local and regional recurrence was 6 months (range 1.2–10.8 and 2.4–38.6 months, respectively) for patients with SCC and for the only patient with basal cell carcinoma who had a local recurrence was 10.8 months.

### 3.3 Regional tumor control, distant metastasis, and survival

Regional recurrence was seen in five patients. One of these patients had a T1N1 disease when diagnosed, was on immunosuppressive drugs due to earlier bone‐marrow transplant for leukemia, and finally died from lip cancer. The other four patients had a regional relapse that could be successfully salvaged with a combination of surgery and EBRT.

One patient with a salivary gland carcinoma had a distant recurrence as first sign of treatment failure, from which he later succumbed.

**FIGURE 3:**
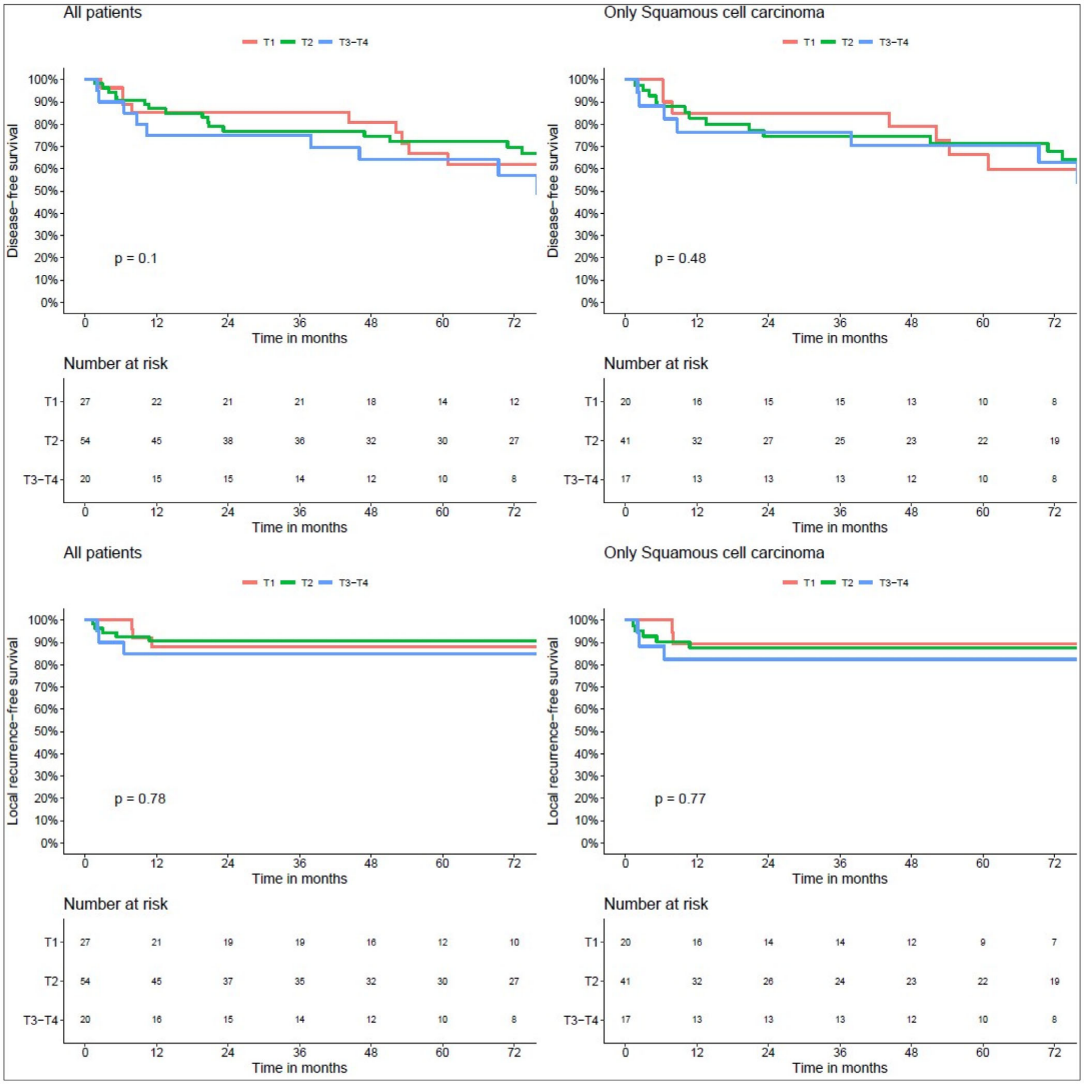
DFS and LFS for all patients and for squamous cell carcinoma by T‐category. [Color figure can be viewed at wileyonlinelibrary.com]

At 3 years DFS was 79% and LFS 89% and at 5 years 69% and 89%. For patients with SCC, DFS at 3 and 5 years was 78% and 70% and LFS 87% and 87%, respectively (Figure 3). Important variables with respect to DFS are displayed in Table 3. Detailed characteristics of all patients with a tumor recurrence can be seen in Table 4. Most patients were old and died from intercurrent disease. Seven patients (7%), six with SCC, succumbed from their lip cancer together with one patient with a salivary gland cancer. Three out of the seven patients (43%) who died from lip cancer had N+ disease at the time of diagnosis.

**TABLE 3:** Statistical analysis of DFS.

Outcome *p*‐value	All patients	Squamous cell carcinoma
Age, <70 vs. ≥70	0.00055	N/A
Sex, female vs. male	0.60	N/A
Oral commissure involvement	0.52	0.27
Tumor classification (T1 vs. T2 vs. T3‐T4)	0.10	0.48
Tumor diameter	0.20	0.66
Tumor grade	‐	0.10
Growth pattern	0.48	0.71
Upfront surgery vs. primary RT	0.50	0.68
Upfront surgery vs. T‐stage	0.00011	0.0089
Upfront surgery vs. tumor diameter	0.0024	0.044
Primary RT vs. T‐stage	0.53	0.65
Primary RT vs. tumor diameter	0.38	0.70

**TABLE 4:** Recurrences with detailed information.

Characteristics														
Patient	Status				Tumor stage					Treatment			Recurrence	
	Age (year)	PS	Alive	DoD	TNM	Tumor diameter (cm)	Histology	Grade	Site	Primary treatment	Radiotherapy dose, BED_10_ Gy	Salvage treatment	First site of recurrence	NED (months)
1	80	2	No	No	T2N0	2.0–3.0	SCC	Moderate	LL	Surgery	62.2	Yes	Local	11
2	68	1	No	No	T4N0	>4.5	SCC	Well	LL + OC	Radiation	87.3	Yes	Local	2
3	66	0	Yes	‐	T1N0	1.5–2.0	BCC	‐	UL + OC	Surgery	78	Yes	Local	11
4	53	0	No	Yes	T2N0	3.0–4.0	SCC	Poor	LL + OC	Radiation	73.5	Yes	Local	2
5	86	2	No	Yes	T2N1	3.0–4.0	SCC	Moderate	LL	Radiation	88.2	Yes	Local	3
6	62	0	No	Yes	T2N1	3.0–4.0	SCC	N/A	LL + OC	Radiation	92.7	Yes	Local	5
7	81	1	Yes	‐	T1N0	≤1.5	SCC	Moderate	LL	Surgery	74.1	Yes	Local	8
8	80	0	No	No	T1N0	≤1.5	SCC	Poor	LL	Surgery	65.3	Yes	Local	8
9	81	2	No	Yes	T3N0	>4.5	SCC	Poor	UL + OC	Surgery	70.2	No	Local	6
10	76	0	Yes	‐	T2N0	3.0–4.0	SCC	Well	LL + OC	Surgery	66.3	Yes	Local	1
11	83	1	No	Yes	T3N0	>4.5	SCC	Well	LL	Surgery	70.2	Yes	Local	2
12	34	0	No	Yes	T1N1	≤1.5	SCC	Poor	LL	Surgery	70.4	No	Regional	6
13	83	2	No	No	T2N0	3.0–4.0	SCC	Poor	LL	Radiation	89.7	Yes	Regional	4
14	81	0	Yes	‐	T2N0	3.0–4.0	SCC	Well	LL + OC	Surgery	70.2	Yes	Regional	26
15	82	0	No	No	T3N0	>4.5	SCC	Moderate	UL	Radiation	46.8	Yes	Regional	38
16	81	2	Yes	‐	T1N0	≤1.5	SCC	N/A	LL	Radiation	70.2	Yes	Regional	2
17	81	0	No	Yes	T3N0	4.0–4.5	SGC	‐	UL	Surgery	87.3	No	Distant	10

Abbreviations: BCC, basal cell carcinoma; DoD, dead of disease; LL, lower lip; NED, no evidence of disease; OC, oral commissure; PS, WHO performance status; SCC, squamous cell carcinoma; SGC, salivary gland carcinoma; UL, upper lip.

### 3.4 Toxicity/aesthetic outcome

**FIGURE 4:**
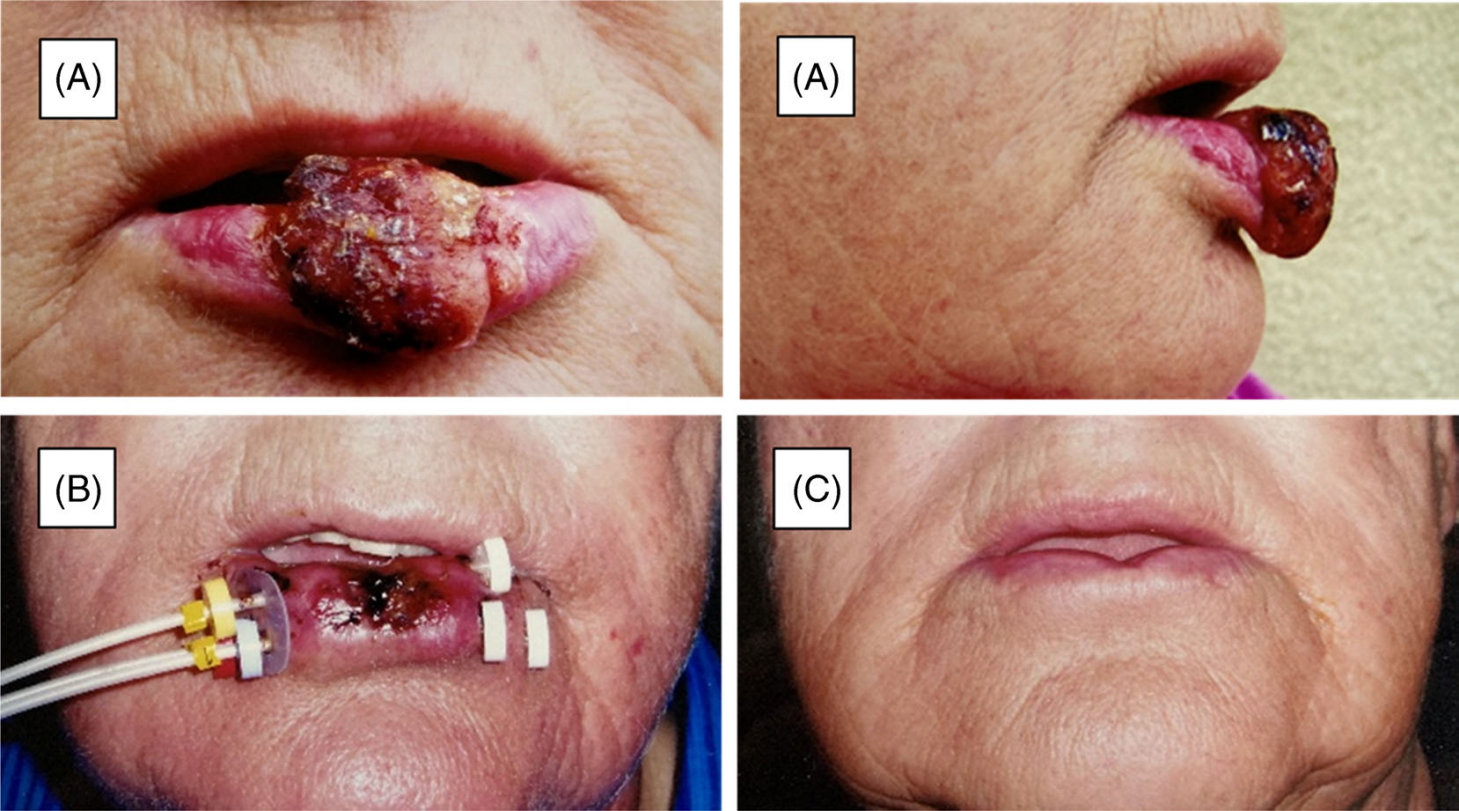
Patient with a T3N0 squamous cell carcinoma. (A) Two days before treatment. (B) Start of brachytherapy. (C) Six months after treatment. Given dose EBRT 50 Gy and HDR‐BT 18 Gy. [Color figure can be viewed at wileyonlinelibrary.com]

Toxicity of treatment was remarkably mild. The first time of follow‐up was at 6 weeks after completed treatment. At this time only 12% grade 1–2 and 6% of grade 3 toxicity remained. There was no documented late toxicity scored >grade 2. No mandibular necrosis was registered. The total dose to the mandible could vary depending on the fractionation schedule. The optimization procedures during the BT planning could mostly keep the 100% isodose outside the mandible and the insertion of at least a 5‐mm sterile gauze dressing between the target volume and the mandible could reduce the dose even more to this structure. Fibrosis grade 1–2 was registered in five patients. There were no patient‐reported limitations with respect to eating, drinking, swallowing, mouth opening, proper insertion of dentures, or speech. Physician‐reported cosmetic outcome for 87 patients was scored as “excellent” in 64 patients, “good” in 13, “fair” in 7, and “poor” in 3 patients. Of those patients labeled as having an “excellent” cosmetic outcome 91% were treated primarily with RT compared to 53% of those with prior surgery. Of those patients treated primarily with the combined regimen 98% had “excellent” or “good” outcome. Out of the 10 patients with a “fair” or “poor” aesthetic outcome, 9 had prior surgery. This means that a bad cosmetic outcome was registered in 9/40 (23%) of patients who had upfront surgery compared to 1/47 (2%) in those who had the regimen as a primary therapy (*p* = 0.003). Example of a patient before, during, and after treatment is seen in Figure 4.

## 4 DISCUSSION

We found combined EBRT and BT resulted in high levels of disease control and good aesthetic outcome for patients with carcinomatous lesions of the lip, not eligible for surgery. The choice between different treatment regimens for lip cancer has for long been dependent on the size of the tumor, its localization, the histopathological subtype of the tumor and the expected function and cosmetic sequelae preferably after primary surgical treatment. The present study reports the results of a treatment program introducing HDR‐BT for patients with malignant carcinomas of the lip as an alternative regimen in order to secure these goals for more patients, that is, to sterilize the tumor in the lip while preserving its function and a good cosmetic outcome. The results represent the experience in one treatment center over a period of about 20 years. All patients in this series had either prior surgery that had failed because of residual or rapidly relapsing tumor or had tumors, usually large, for which surgery was considered inappropriate. No patient with prior surgery was given their RT as an adjuvant procedure. Most patients had medium‐sized or large tumors, both with regard to T category, but also when it comes to size of the tumor within respective T category. Therefore, patients in the present cohort could be considered “high‐risk patients” with less good prognosis. The bulk of patients in the present series presented with squamous cell carcinomas, close to 80%. Even if intrinsic radiation sensitivity is not considered equal for the different tumor types represented in the study, with basal cell carcinoma being somewhat more radiosensitive and salivary gland carcinomas more radioresistant, there is a large variation with respect to this factor among individuals within the different histological subtypes. Other radiobiological factors such as hypoxia and proliferation of tumor cells before and during radiotherapy could influence treatment outcome. However, there are no data in the literature indicating differences with respect to these characteristics of the tumor subtypes seen in the present study. Therefore, it could be argued that the same basic RT schedule could be used irrespective of tumor type with the aim of sterilizing the local tumor. The results in the present study do not contradict this hypothesis.

Before the introduction of this regimen in our institution, it was a common impression that surgery, as used for most T1 tumors, was often not radical. This impression was supported by findings in the literature that local recurrence after primary surgery for tumors >1.0 cm occurred in 10%–15% of cases, depending on tumor size.^11^, ^12^ Moreover, other studies showed that up to one third of patients treated with upfront surgery can be left with very close (<5 mm) or positive margins, which constitutes a major factor for local failure together with tumor size, pathological grade, and perineural invasion of tumor.^13^, ^14^ The primary aim of surgery is tumor resection with a clinical clearance of ideally 0.8 cm according to guidelines of the Swedish National Care Program for Head and Neck cancer, and even 1.0 cm for lesions more advanced than category T1.^1^, ^15^ Since the lower lip is only 6–7 cm, the removal of a 1.0‐cm T1 tumor could mean that almost half of the lip must be excised to fulfill the criteria for a radical resection. In order to avoid a reconstructive procedure with the risk of functional deficits, it is understandable that several primary resections turn out to be insufficient, leading to the need for either further, potentially mutilating surgery or RT. To use ultrasound to measure more precisely the extension of the tumor might be a possibility to avoid inadequate surgery.^1^

For moderately advanced lesions, T2 tumors of 2–4 cm, removal of greater than half of the lower lip with simple closure produces an even higher risk of a poor cosmetic and functional result. In a recent study from an academic center for plastic surgery where most patients had small tumors, 91 out of 104 had T1 and 12 patients T2 tumors, 21 reported loss of sensation, causing discomfort and/or functional problems. Twenty patients had problems with normal oral functions such as chewing or proper use of dental protheses after surgery and 14 reported drooling problems.^16^ The surgical resections that require V‐ or W‐shaped excisions or flaps may lead to microstomia and limited mouth opening. Therefore, a reconstructive procedure is almost always considered unavoidable. The reconstructed lip may look normal in a photograph but lacks often both sensory and motor innervation as well as elasticity. Wedge excision with primary closure and the stair‐step technique is often used for small tumors whereas an Abbe flap or a modified Bernard‐Fries technique is used for larger tumors. The latter techniques have been reported to impact negatively on important functions of the lip with hypaesthesia, pronunciation, and mouth opening.^17^ Moreover, patients treated with multiple interventions have reported significantly more functional problems than patients treated only once, which suggests that a previous inadequate resection influences negatively on quality of life.^18^, ^19^, ^20^ This finding emphasizes the need for initial treatment in a specialized center where several treatment modalities are available. So, even if many local failures can be salvaged, the treatment of a local lip recurrence is potentially associated with more long‐term sequelae.

Several patients in the present study had large volume tumors in their respective T category. A T1 tumor can be a few millimeters up to 2 cm and a T2 tumor from 2 to 4 cm which may influence both the selection of patients for surgery as well and curability. More than half of all tumors, 52% were >3 cm, and in the group of patients with squamous cell carcinoma 59% of tumors were so extensive. Twenty patients with a T1 tumor had curatively intended surgery before RT (74%), and for patients with T2 and T3/T4 tumors, corresponding figures were 21 (39%) and 4 (20%), respectively. In the group of 78 patients with squamous cell carcinoma, 29 patients (37%) had prior surgery, 14 (70%) for T1 tumors, 12 (29%) for T2, and 3 patients (19%) for T3 tumors.

The results in the present study with a high rate of local tumor control in combination with mild long‐term morbidity are challenging. The results are in line with those of other researchers but the patient material in the present analysis is characterized by advanced tumors, that is, with many T2‐T3 tumors with large sizes within respective T category and often growing close to the oral commissure. However, 11 out of 101 patients experienced a local failure, with half as many failures in the group of patients who had the combined radiotherapy protocol upfront compared to those who started with curative surgery. The four patients in the former group had advanced tumors, one patient with a T4 tumor, and the other three with large T2 tumors ranging from 3.0 to 4.0 cm in longest diameter, while tumors in the latter group were remarkably smaller with three out of seven tumors in category T1. The reason why more patients with prior surgery, developed more local failures despite the prior debulking procedure, can only be speculated upon: these patients may have had biologically more aggressive tumors but it is also likely that the surgical procedure makes the tumor tissue more radioresistant, inducing both hypoxia and an increased proliferation of residual clonogenic tumor cells.

Considering the low registered rate of toxicity in the lip it is reasonable to speculate that higher RT doses should be prescribed for certain patients, such as those with large tumors, or those with residual tumor after prior surgery, irrespective of tumor size. On the other hand, there was no correlation in the present series between delivered RT dose and rate of local recurrence (Figure 1). Moreover, the dose levels used in the present study are in line with those described in other studies in the literature.^4^, ^5^, ^21^, ^22^, ^23^, ^24^

To decrease risk of local recurrence by adding chemotherapy given concomitantly with RT does not seem attractive for this mostly elderly patient population. However, in a recent pilot study involving patients with squamous cell carcinoma of the skin, a high percentage of patients had a pathological complete response with minor toxicity after treatment with two doses of neoadjuvant programmed cell death protein 1 inhibitor before surgery.^25^ This finding is interesting with respect to lesions with similar pathology in the lip. When given before RT it could shrink the tumor and therefore facilitate adequate coverage of the tumor volume with the BT catheters and improve RT dose distribution but also treat spread disease.

The aesthetic outcome was impressive for most patients. However, it was remarkably worse for patients who had prior surgery since 9 patients out of 10, who had a fair or poor outcome belonged to that group of patients. Moreover, this was also true for patients with T1 tumors. The good aesthetic outcome was paralleled by the low rate of treatment morbidity from normal tissues. This advantage of the treatment regimen might be influenced by the combination of EBRT and BT where the potential negative impact of the high‐dose 150% and 200% volumes of BT is diminished when the intended curative doses are reached. The presented data support the use of the described combined regimen for several patients diagnosed with a lip carcinoma. Our results should in theory deserve multicentric comparative and prospective evaluations which might be difficult since BT is available nowadays in only large treatment centers.

The present study has limitations, the retrospective study design and the use of different total doses and fractionation schedules, deviating from the standard protocol, considered appropriate due to patients' and tumors' individual characteristics. However, this condition describes a learning period in a main treatment center when a new regimen is introduced and therefore represents “real world data.” Moreover, all patients were consecutively included and had a prospectively complete long follow‐up regarding tumor control and side effects. It is also realized that the evaluation of cosmetic outcome is physician and not patient‐reported. This might possibly confer a bias to the final results and their interpretation.

## 5 CONCLUSIONS

Data in the present report support the use of a combined regimen of EBRT and BT as primary treatment for patients with lip cancer, most notably for those with T2 and T3 patients but also for those with large T1 tumors. Even if surgery remains the mainstay of therapy for a T1 lip cancer such tumors should not be treated without a meticulous definition of tumor size considering the extension of a normal lip of only 6 cm and the need for adequate surgical margins. Data in the literature indicate that similar results as in the present series could be achieved with only BT. However, the combination of HDR‐BT with EBRT as described in the present regimen might have advantages when it comes to side‐effects and aesthetic outcome.

## CONFLICT OF INTEREST STATEMENT

The authors declare no conflicts of interest.

## ETHICS STATEMENT

The study was approved by Swedish Ethical Review Authority.

## Data Availability

The data that support the findings of this study are available on request from the corresponding author. The data are not publicly available due to privacy or ethical restrictions.
